# BENEFITS OF SERVICE LEARNING FOR STUDENT PARTICIPANTS AND OLDER ADULT RECIPIENTS

**DOI:** 10.1093/geroni/igae098.2946

**Published:** 2024-12-31

**Authors:** Krista Cline, Catherine Bain

**Affiliations:** Butler University, Indianapolis, Indiana, United States; University of Oklahoma, Norman, Oklahoma, United States

## Abstract

It is clear that service learning benefits college students, while also benefiting the community. While research on intergenerational service learning has focused on the benefits for the students, very few studies have focused on the older adults who are the recipients of the service learning. For the current study, we were interested in the benefits of service learning for both the college students and the older adults who participated in a service learning course. Data for the current study is qualitative and were collected from both the students in a sociology of aging and the life course service learning class (n=22), and the older adults who participated as recipients of the service learning (n=22). Data from the students was collected via student journals and open-ended questionnaire responses written by the students. Data from the older adults were collected via interviews by the students as well as open-ended questionnaire responses written by the older adults. The following themes emerged as benefits to students; (1) a better understanding and less fear of aging; (2) a desire to learn more about older adults; (3) a desire to engage more with older adults. The themes for the benefits to the older adults included; (1) improved social connections and companionship, and; (2) becoming family. We found that engaging in intergenerational service learning courses is beneficial to all those who are involved.

